# Successful Implementation of Menstrual Cycle Biomarkers in the Treatment of Infertility in Polycystic Ovary Syndrome—Case Report

**DOI:** 10.3390/healthcare11040616

**Published:** 2023-02-18

**Authors:** Aleksandra M. Kicińska, Aneta Stachowska, Anna Kajdy, Tomasz H. Wierzba, Radosław B. Maksym

**Affiliations:** 1 Department of Physiology, Faculty of Medicine, Medical University of Gdansk, ul. Debinki 1, 80-210 Gdańsk, Poland; 2 1st Department of Obstetrics and Gynecology, Centre of Postgraduate Medical Education, ul. Żelazna 90, 02-004 Warszawa, Poland

**Keywords:** polycystic ovary syndrome, progesterone, menstrual cycle, fertility awareness methods, fertility biomarkers, case report, luteal phase

## Abstract

Polycystic ovary syndrome (PCOS) is the most common cause of anovulatory infertility. Absent, impaired, or rare ovulation induces progesterone deficiency in the luteal phase, which is a critical problem in PCOS. A usual pattern of progesterone administration from a fixed and arbitrary pre-determined day of a menstrual cycle may preserve infertility but can easily be avoided. We present the case of a 29-year-old infertile woman who had been ineffectively treated for over two years. We introduced a line of therapy that was suited to her individual menstrual cycle by implementing biomarker recording. Supplementation based on a standardized observation of the basal body temperature (BBT) and cervical mucus stopped the vicious circle of absent ovulation and hyperandrogenism, restoring regular bleeding, ovulation cycles, and fertility. The implementation of a reliable fertility awareness method (FAM), accompanied by a standardized teaching methodology and periodic review of the observations recorded by the patient, validated through an ultrasound examination and plasma gonadotropins, estrogens, and progesterone concentrations, is key to achieving therapeutic success. The presented case is an example of a clinical vignette for many patients who have successfully managed to improve their fertility and pregnancy outcomes by applying the principles of a personalized treatment approach together with gestagens by recording their fertility biomarkers.

## 1. Introduction

Polycystic ovary syndrome (PCOS) is the most common endocrinopathy and systemic metabolic disorder among patients who are seeking infertility treatment. In young women, the symptoms include a disturbed menstrual cycle and ovulation, infertility, acne, or hirsutism due to hyperandrogenism. At the same time, metabolic changes towards obesity, insulin resistance, an increased risk of type 2 diabetes mellitus, dyslipidemia, and metabolic syndrome are also present. They are frequently linked to an increased risk of cardiovascular diseases, endometrial cancer, and mental and immune disorders [1,2,3]. 

PCOS is one of the most frequently mentioned causes of infertility accounting for up to 56% of cases [4,5] and it is affecting more and more women worldwide. According to the latest reports, about 8-13% and even from 4% to 26% of women of reproductive age worldwide are affected by it [6,7]. An assessment of PCOS prevalence depends on which population of women the estimates were collected on and the diagnostic criteria used by the researchers. So far, the criteria of the National Institute of Health (NIH) [8], followed by the Rotterdam criteria [9] and then by the Androgen Excess Society [10], have been used chronologically. The current recommendations point to the Rotterdam consensus as the most clinically useful in diagnosing PCOS, considering the need to distinguish between individual phenotypes of this syndrome [9]. The prevalence of PCOS continues to increase. Human reproduction studies published in 2017 on the prevalence of polycystic ovary syndrome (PCOS) on global, regional, and national levels were carried out in 194 countries and territories in terms of age and socio-demographic index. The authors reported that over 1.5 million women of childbearing age worldwide experience PCOS [11]. The results of PCOS prevalence analysis in 204 countries and territories in 1990–2019 were published in 2022. In 2019, age-standardized global point prevalence surveys and annual PCOS incidence rates had increased by nearly 30% since 1990 [12]. However, according to the Global PCOS Treatment Market’s Forecast and Opportunities report, the prevalence of PCOS may reach approximately 5.1 million by 2025 [13]. The wide spread of epidemiological data on this syndrome is primarily because the guidelines for PCOS diagnostic procedures are often unclear and inconsistent, even for healthcare professionals. For this reason, it is believed that up to 70% of women with PCOS remain undiagnosed [14]. Several pathophysiological models are considered in PCOS, assuming that the primary disorder is the main cause of this endocrinopathy. Both insulin resistance and hyperandrogenemia, as well as chronic inflammation and increasingly discussed genetic and epigenetic background, are the basis for the discussion around the primary cause of PCOS. Regardless of the knowledge of PCOS pathophysiology, it is natural that the clinical picture is dominated by anovulation or rarely occurring ovulation, which generates chronically low progesterone levels in these patients. Progesterone not only ensures the cyclic exfoliation of the endometrium and the occurrence of menstruation but, above all, is an immunomodulatory factor. It not only affects reproduction but also the entire body and women’s health in general [15]. PCOS is characterized by a central dysregulation of the hypothalamic–pituitary–ovarian (HPO) axis with a rapid pulsation of the gonadotropin-releasing hormone (GnRH), followed by a rapid pulsation of the luteinizing hormone (LH). This, in turn, impairs FSH-dependent follicular growth and generates anovulatory cycles with chronic progesterone deficiency [16]. Therefore, a luteal phase insufficiency with all of its clinical consequences is routinely observed among women with PCOS. Thus, treatment with progesterone seems to be justified not only in PCOS but in any situation where luteal phase failure (LPD) occurs. Progesterone physiologically slows down the GnRH and LH impulses and contributes to the proper functioning of the HPO axis. It has been shown that with normalized pulses of central GnRH/LH secretion, excess androgens and hyperinsulinemia will return to normal and the physiological balance between estradiol and progesterone in a woman’s monthly cycle will be restored [17,18]. The latest ASRM guidelines on antidepressants concluded that infertile women with a suspected luteal abnormality due to an underlying disease should be evaluated and treated appropriately to identify the basal abnormality. As for the legitimacy of progesterone administration, the position of the ASRM is still open [19]. However, there are several studies supporting the need for progesterone, experience, and good clinical practice, which show that the cyclic administration of progesterone can have a beneficial effect on women’s reproductive health, pregnancy outcomes, and overall well-being [15,17,18,20,21,22,23].

According to the physiology of fertility, progesterone is synthesized by the corpus luteum in the second phase of the menstrual cycle and blocks the onset of the next ovulation. This knowledge has been used for many years in various methods of observing the female menstrual cycle and determining the phases of fertility and infertility [24]. Different forms of fertility recognition rely on observations of the clinical changes in a woman’s body to determine the estrogen and luteal phases. Among the commonly known symptoms observed in these methods are the variations in mucous secretions, a basal body temperature (BBT) rise, and changes in the cervix consistency. Such methods are increasingly being used to avoid or achieve pregnancy. These methods involve various applications that easily and conveniently allow women to track the changes in their fertility biomarkers and the course of the cycle. Only the applications that are based on scientific evidence and use fertility awareness methods (FABMs) rather than calendar methods can be considered as reliable fertility recognition tools [25,26]. Most FABMs allow women to identify their presumed ovulation and track the phases of the menstrual cycle through daily observations recorded on paper or electronic cycle charts [27].

For each evidence-based method (Billings, Creighton, sympto-thermal, sympto-hormonal, or LAM-lactational amenorrhea) there are levels of evidence in studies that, when used correctly, demonstrate that they are as effective as any commonly used forms of contraception [28] and the rate of unintended pregnancies varies between 1 and 3% in both industrialized and non-industrialized countries [29].

Infertile couples can use FABMs to achieve pregnancy using so-called targeted intercourse. In addition, doctors can use the information from the FABM charts to diagnose and treat cycle disorders and infertility using a personalized medicine approach focused on restoring the normal functions of the reproductive and endocrine systems. However, FABMs are more effective and credible when conducted by a trained instructor [27].

Most patients suffering from PCOS experience disturbed menstruation, demonstrated by irregular, extended cycles and abnormal withdrawal bleeding. Absent or impaired ovulation results in chronic progesterone deficiency. Regarding the standard 28-day period, progestagens are administered from Day 16 or 17 of the menstrual cycle in routine clinical practice, as Days 13-14 are assumed to be putative days of ovulation [30]. However, early progestagen administration disturbs the hypothalamic–pituitary–ovarian (HPO) axis, which can block or hinder upcoming ovulation [31]. Therefore, it is vital for PCOS patients undergoing infertility treatment to have progestogen substitution implemented at the correct time in the second phase of the menstrual cycle. We present a case of a patient with PCOS in whom the introduction of progestogen therapy in line with the individual menstrual cycle pattern restored the regularity of menstrual cycles and was followed by gestation.

## 2. Materials and Methods

A 29-year-old woman was referred to an outpatient clinic after several unsuccessful attempts to achieve pregnancy over a period of two years. The patient and her husband had been diagnosed earlier in another infertility clinic. The patient’s uterine tube patency examination and husband’s semen tests did not reveal any pathology. PCOS was diagnosed based on ultrasound examinations revealing a characteristic morphology of the ovaries, extended menstrual cycles lasting up to several months, and the absence of ovulation. The patient was treated for hypothyroidism and disturbed menstrual cycles by an endocrinologist. The body weight was correct, with a BMI of 19. The patient received 25 mcg of levothyroxine daily and progesterone (100 mg BID vaginally) for 7–10 days from Day 16 of the menstrual cycle. As a result of the implemented treatment, the menstrual cycles became shorter and uterine bleeding occurred regularly every 26–32 days. Subsequently, clomiphene citrate was repeatedly administered to stimulate ovulation but the patient failed to conceive. In vitro fertilization (IVF) was proposed to the couple but they refused for philosophical reasons.

Upon admission to our center, the patient was instructed to have blood tests performed on Day 3 of the cycle to evaluate the luteinizing hormone (LH), follicle-stimulating hormone (FSH), β2-estradiol (E2), anti-Müllerian hormone (AMH), prolactin, thyroid-stimulating hormone (TSH), and free thyroxine (FT4). Additional diagnostic tests were carried out: serum androgen concentration, thrombophilia profile test, and 75 g oral glucose tolerance test (OGTT). To ensure a confident prediction of ovulation based on biomarker observations, the patient was referred to a professional instructor to learn the standardized recording of menstrual cycles. This allowed progestogen to be administered at least three days after the basal body temperature (BBT) increase or change in the morphology of the cervical mucus identified on the basis of observations of vaginal discharge as described earlier [32]. The cycle observation method developed by us is based on the previously known and used FABM fertility biomarkers, such as cervical mucus secretions and basal body temperature measurements (BBT) [27]. An undoubted advantage that sets it apart from other FABMs is the precise monitoring and recording of mucous discharge. The creation of a photo gallery in the form of a mucus secretion picture dictionary allows a detailed description to be made of the clinical condition of a patient’s reproductive tract. The “Pictionary” was created based on collected material consisting of over 2000 vaginal discharge photos from 429 cycle observation charts of our patients, which were compared and arranged accordingly to create a standardized and unified observation and recording model. The cervical mucus changes can be charted with great precision on the cycle observation chart developed by us because, during meetings with a trained instructor, the patient can compare her own photos of vaginal secretions taken during daily observations with the images collected by us in the pictionary “Biomarkers of fertility. An InVivo method”. This serves as the basis of therapeutic recommendations of the physician interpreting the patient’s cycle observation charts, which can, for example, include microbiological cultures from the genital tract due to the presence of an abnormal discharge marked by the patient in the yellow field according to the standardization guidelines provided in our monograph. In addition, a correct interpretation of the BBT curve in conjunction with the development of the mucus cycle contributes much more to the patient’s clinical history than just observing one of the bioindicators [32]. As can be seen on the last chart, also in the case of our patient, charted abnormal mucus observations and concomitant symptoms of discomfort led to a recommendation of a taking a culture from the cervix and the implementation of treatment following the antibiogram. After a BBT rise was determined according to the charting rules, progesterone was administered correctly without blocking ovulation. 

The approval of the Bioethical Committee was waived for this study due to the statute and regulations of the local Research Ethics Committee at the Centre of Postgraduate Medical Education in Warsaw, Poland, which did not give approval for case reports and retrospective non-interventional studies. We obtained the patient’s consent to publish her case, test results, and cycle observation charts after anonymization.

## 3. Results

**Figure 1:**
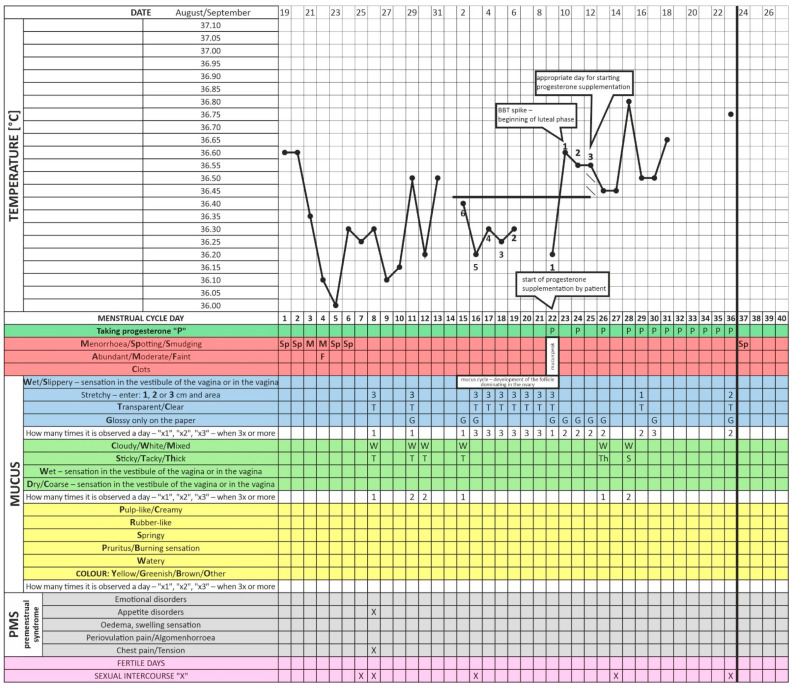
Menstrual cycle observation chart at the time when the patient started taking progesterone incorrectly—too early—(i.e., on Day 22 of the menstrual cycle).

**Figure 2:**
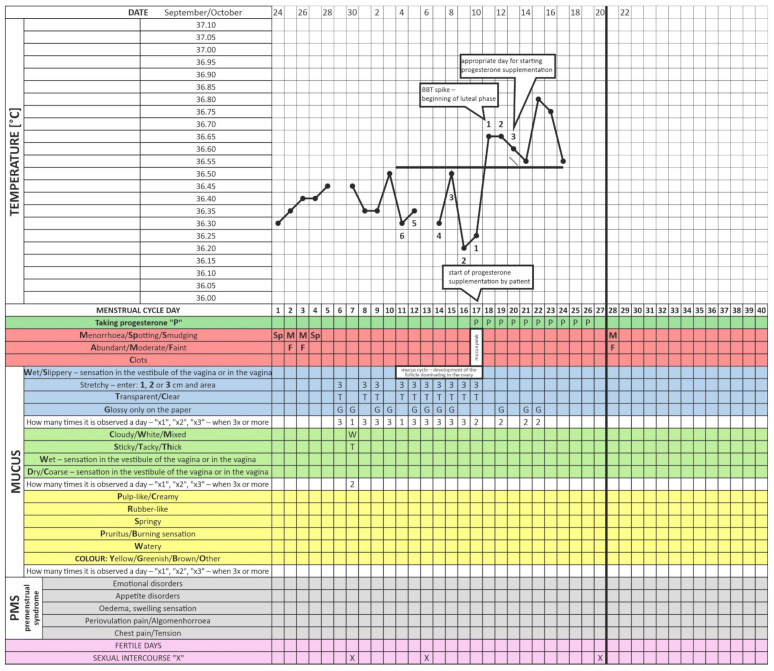
Menstrual cycle observation chart at the time when the patient started taking progesterone incorrectly—too early—(i.e., Day 17 of the menstrual cycle).

Initially, the patient began her observations but, based on previous recommendations from another clinic, did not introduce progestogen therapy on the right day after an ovulatory event (Day 3 after the BBT rise) (Figure 1 and Figure 2). After six months of menstrual cycle observations without direct instructor supervision, the patient finally consulted the center’s infertility specialist physician for follow-up. A physical examination revealed androgenization in the form of hirsutism (score nine on the Ferriman–Gallwey scale), hyperkeratosis of the elbow and knee epidermis, and acanthosis nigricans in the axillary area. Laboratory tests revealed a TSH level of 2.5 mU/L, which is the upper limit of the recommended preconception level, a high insulin plasma concentration at 60 min, and reactive hypoglycemia in 75 g OGTT (fasting; 60 and 120 min after glucose loading; the insulin and glucose concentration amounted to 4.4/52/23 U/L and 78/106/67 mg/dL, respectively). Due to the reported discomfort and vaginal discharge that suggested a reproductive tract infection, a cervical canal culture was obtained. The culture revealed a significant growth of Streptococcus agalactiae. Even though the microorganism is considered to be commensal of the female reproductive tract, targeted antibiotic therapy (amoxicillin) was prescribed because of the patient’s symptoms.

**Figure 3:**
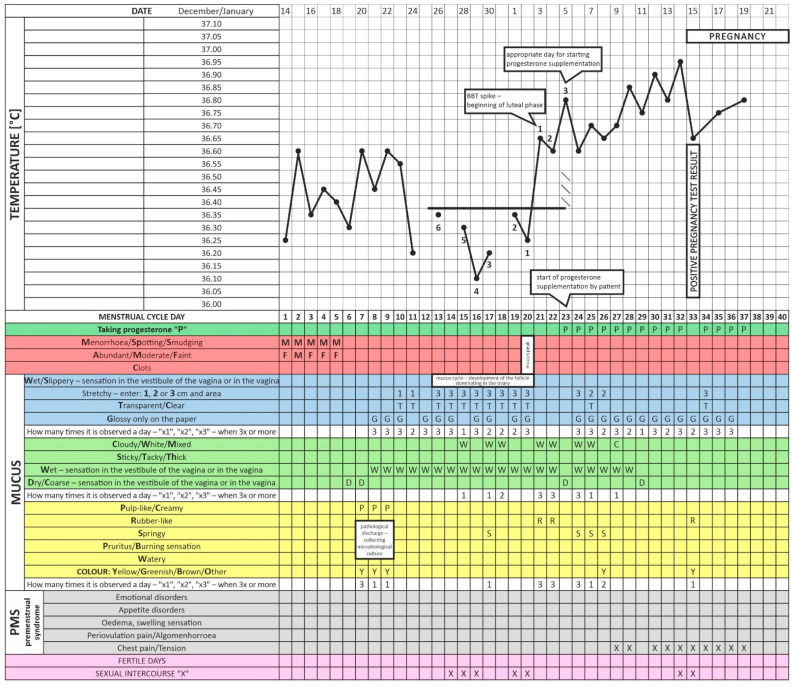
Menstrual cycle observation chart at the time when the patient started progestagen (Dydrogesterone) therapy correctly (i.e., on the third day of increased BBT (the patient conceived in this cycle)).

Moreover, the patient observed marked abnormal mucus for several months in the yellow field of pathological discharge on the menstrual cycle observation chart. In addition, the blood tests revealed an increased risk of thrombosis. The activated protein C resistance (APC-R) ratio of 1.9 was below the standard, suggesting inherited thrombophilia. Free protein S was below the standard value of 55%. To optimize the TSH level, the levothyroxine dose was increased by 25 mcg. A low-glycemic diet was introduced due to an incorrect OGTT. To ensure that the recommended progestagen therapy was started on the right day, the patient had regular consultations with an instructor trained in teaching menstrual cycle observation charting. Progestogen supplementation was introduced in the next cycle, on Day 23, three days after the BTT increase (Figure 3). Biochemical and ultrasound examination confirmed that gestation occurred in the patient for the first time in her life in the reference cycle. Plasma β-HCG 265 U/L and 368 U/L were present on Days 16 and 17 of the elevated BBT, respectively, while the presence of a gestational sac in the uterine cavity was confirmed by an ultrasound exam on Day 48 of the cycle. Due to the suspected thrombophilia, a prophylactic dose of 40 mg sc. low-molecular-weight heparin (LMWH) was included. Progestogen therapy (Dydrogesterone; Duphaston, Abbott; 3 × 10 mg daily) was continued until the 12th week of pregnancy. The labor was spontaneous and the baby was born healthy in Week 40 of gestation, with a birth weight of 3.245 g and an Apgar score of 10. 

## 4. Discussion

Oligo/anovulation in PCOS patients results in a chronic progesterone deficiency. Thus, progesterone has a powerful inhibitory effect on gonadotrophin-releasing hormone (GnRH) and luteinizing hormone (LH) secretion. Elevated GnRH and LH release is followed by an excessive stimulation of theca cells. Consequently, ovarian androgen production is upregulated, promoting a vicious circle of ovulation suppression [16,22,33]. The current PCOS management recommendations involve hormonal contraceptives to control irregular menstruation and hyperandrogenism symptoms [34]. Ovulation is either completely absent or occurs rarely in chronic progesterone deficiency. Progestogen administered regularly for several menstrual cycles can restore normal GnRH action, normalize FSH and LH secretion and ovarian follicle growth, and ameliorate proper ovulation. Moreover, supplementation with progesterone can resume optimum endometrial receptivity for embryo implantation, providing a beneficial immunomodulating influence on the immune system [22]. The suppression of LH levels and the related ovarian androgen production reduces the hyperandrogenism and plasma dihydrotestosterone (DHT) concentrations [16,22,35]. However, the precise control of metabolic disorders is time- and effort-consuming. The reduction in insulin resistance by following an appropriate diet, introducing key lifestyle changes, and the administration of pharmacological preparations such as metformin or inositol often requires following strict regimens for several months [22,36]. Most patients with luteal phase insufficiency or PCOS routinely use progestogens after Day 16 of the menstrual cycle, regardless of the time of ovulation. This is despite the fact that it is more efficient to start the administration of gestagens on a specific day identified on the basis of signs of ovulation revealed by ultrasound. Nonetheless, women often find it challenging to frequently perform ultrasound monitoring or to have their blood hormone levels tested due to the logistical, economic, mental, and social constraint thereof [37]. 

LH home ovulation kits are often unreliable in PCOS patients because of the excessive LH secretions that remain unrelated to ovulation. It should be emphasized that the high diagnostic and treatment costs and limited access to laboratory tests or specialist physician consultations are among the most significant barriers for persons who experience infertility. Hence, there is an urgent need for inexpensive, readily available, and reliable solutions. The observations of such fertility biomarkers such as the cervical mucus discharge or the BBT measurements are seldomly used in reproductive practice, which can be attributed to the FABMs being widely regarded as being of little use, unreliable, and burdened by significant errors due to the absence of user discipline, erroneous effects resulting from everyday life, stress, comorbidities, coexisting chronic diseases, and acute infections [12,37]. 

FABMs have, nevertheless, been reported to improve the female reproductive cycle and fertility with minimal risks of adverse effects and low costs [37]. The reliable use of FABMs requires standardized charts for recording the patient’s biomarkers and all possible disturbances in the cycle to be taken into consideration. We aimed to develop a method based on the BBT and cervical mucus observations that is convenient for the patient and helpful for the physician, assisting them in their regular fertility practice, and also offering the opportunity to educate patients and ensure their active participation in the therapeutic process [32]. 

It has been well documented that a BBT increase is related to a progesterone surge right after ovulation due to the hormone being produced by the corpus luteum. Moreover, we also noticed that the shape of the BBT curve can be indicative of luteal phase disturbances. Specific features of cervical mucus observed in the first phase of the menstrual cycle represent the estrogen-induced vaginal discharge. Not only is a single or double assessment of the serum hormone levels or their derivatives more expensive but they can fail to demonstrate upcoming ovulation due to their significant momentary fluctuations in the blood. This is why a professional observation of the signs of fertility can prove to be more useful and provide more extensive information when it comes to deciding upon the suitable clinical management of an infertile patient than an assessment of reproductive hormone plasma concentrations and occasional ultrasound monitoring. In addition, the same woman can have significant inter-cycle fertility variability [38]. Each subsequent cycle may look different due to endogenous and exogenous factors. The change in mucus discharge from fertile to infertile (referred to as the PEAK of the fertile mucus signs) and the accompanying BBT spike confirming ovulation will not always take place on the same day of the menstrual cycle in the same woman. Therefore, the 24 h home observations and charting of the menstrual cycle conducted by the patients based on the pictionary and training given by a trained instructor may provide much more detailed information on the course of the relevant cycle phases and clinical symptoms, depending on hormonal fluctuations or microbiological and immunological events, than a simple and selective gynecological and ultrasound examination or blood test. Only a combination of all of the available methods used in everyday clinical practice concerning the standardized observations of an individual and their currently ongoing cycle can help to make an accurate diagnosis and lead to an application of the most targeted and personalized treatment.

In the FABM developed by our group, the patient, under the guidance of a qualified instructor, charts the BBT and cervical mucus observations by comparing them to the vaginal discharge photos in the pictionary created by us. The instructor teaches the patient how to correctly interpret the observed discharge and measure the BBT, and corrects any charting errors, assisting them in implementing and following the charting rules. We compared the cervical mucus photos taken by our patient on specific days of the menstrual cycle with the reported clinical data. Pathological mucus secretion can negatively influence the transport of spermatozoa in the reproductive tract and their capacitation—both aggravating infertility [39]. Considering the involvement of systemic diseases and infections, ultrasound monitoring, blood hormone tests, and microbiological cervical swabs were also performed [32]. 

As a standard, BBT is measured under the tongue using an electronic thermometer in conditions corresponding to the basal metabolic rate (BMR) after at least 1–3 h of sleep. The corresponding observations of the cervical mucus and its changes over the course of a menstrual cycle, the follicular phase and the progesterone-dependent luteal phase, help to determine the time of ovulation. Unexpected situations and incidents disturbing correct temperature measurement only affect the interpretation of the entire temperature curve if they last for a maximum of 2–3 consecutive days.

The standardized charting of the two fertility biomarkers, BBT and cervical mucus, allows ovulation to be determined more accurately than by using BBT or mucus observations alone. A BBT rise, coinciding with a mucus surge, namely the last day of the estrogen-dependent mucus with the most fertile characteristics, helps to determine the period of ovulation and the border between the two phases of the patient’s menstrual cycle [40].

In addition, the careful observation of the mucus in these methods and the ability to interpret the morphology of the observed secretions as normal or pathological allows for the microbiological diagnosis of the cervical canal to be started. The colonization of pathogenic microbes can induce humoral cross-reactions between microbial and sperm antigens. This situation may suggest idiopathic infertility caused by sperm capacitation disorders [41] and cause clinically overt or subclinical endometritis via the ascending route, generating problems with implantation.

The enclosed charts demonstrate how progesterone supplementation by the described patient from Days 17–19 of the cycle (without taking fertility biomarkers into account) may disturb ovulation and impair fertility. Although it caused regular endometrium exfoliation, it also prevented gestation (Figure 1 and Figure 2). This patient followed such a procedure for about two years [42]. We present a clinical situation where progestagen was administered for the first time according to fertility biomarkers three days after the relevant BBT increase (in the luteal phase of the cycle, Figure 3). This did not occur on Days 17–21 as it previously did but on Day 23 of the cycle. Note that delayed ovulation is extremely common in patients with PCOS. The too-early administration of progesterone disturbs the HPO regulatory axis, inhibits the LH surge, and consequently disturbs and modifies ovulation. This is why the routine use of progesterone from a pre-determined day of the cycle (without considering the patient’s individual and ever-changing menstrual cycle) can consolidate persistent fertility disorders, as in the studied case. Our data indicate that therapy tailored to a specific person’s menstrual cycle biomarkers seems to be an invaluable and highly reliable treatment for infertility and menstrual cycle disorders [32]. Cyclic progesterone therapy appears to be the most physiological and effective therapy for luteal phase deficiency (LPD). Despite being evidence-based and easily applicable in clinic settings, it still requires further research and translation into the knowledge of health professionals and women themselves [18]. The described case and the concept of adjusting the administration of progesterone to individual needs is only one of many analogous cases from our clinical practice. We wanted to exemplify this seemingly trivial but, on the other hand, too common a therapeutic problem. 

We know that the description of a single case is a particular limitation in scientific inference. On the other hand, in addition to using progesterone at the right time, therapy was carried out in parallel to compensate for the identified disorders in this woman. However, since we use such a treatment regimen as a standard and it also works well on other patients, we are convinced that this is the right procedure and approach to take. In previous cycles, the patient also underwent other therapies, such as thyroid treatment. Despite this, however, pregnancy did not occur because, as we wrote above, progesterone was administered according to calculations and not in line with the current course of the cycle and, most importantly, after ovulation. In the cycle in which pregnancy occurred in this patient, an ultrasound was not performed to monitor the development of the follicle and the occurrence of ovulation. However, the ultimate confirmation of the occurrence of ovulation is the pregnancy that we had just achieved in this cycle when progesterone was turned on later than usual, but according to the observations of fertility bioindicators, was late as on Day 23. The patient had taken progesterone on an arbitrary day of the cycle for more than two years and had failed to conceive. The cyclic progesterone treatment that lasted for two years promoted the monthly shedding of the endometrium and improved the endocrine profile. These facts demonstrate that it was easier for a patient with PCOS to ovulate spontaneously but pregnancy did not occur because the second phase hormone was switched on before the completion of follicle development, effectively blocking its rupture and preventing possible conception.

Too early an administration of progesterone results in the blockage of FSH release by the pituitary gland and inhibits the further development of the follicle. Knowledge of the product of the mucus cycle observed by the patient, which reflects the follicle’s growth and maturation to ovulation, allows inferring what is currently happening in the ovarian cycle. On the way to ovulation, the granulosa cells of the follicle produce more and more E2, which stimulates the cervical crypts to increasingly secrete fertile-type mucus. The observed increase in the amount of mucus and the improvement in its quality due to increasing concentrations of estrogen in the ovary reveal a characteristic development of the mucus cycle as charted on the chart. If it ends with a persistent rise in BBT and a sudden change in mucus morphology to a thick mucus due to the release of progesterone, we have clear evidence of the presumed ovulation [38,43]. The final clinical proof of regular ovulation, that is, the complete rupture of the luteinizing follicle wall, accompanied by the release of a mature and competent oocyte, is pregnancy alone. 

There is no evidence to suggest that the cyclic administration of progesterone restores ovulation, but in the case of PCOS, ovulation can be stimulated or can occur spontaneously. However, it should be borne in mind in everyday practice when prescribing progesterone treatment that too early an administration of progesterone in the follicular phase may impair follicular development. Progesterone suppresses the preovulatory LH surge by interrupting the activation of kisspeptin neurons in the ventral atrioventricular nuclei (AVPV) that leads to the GnRH/LH surge. Menstrual cycle observations and proper progesterone switching help to protect against such iatrogenic contraception/infertility. Paradoxically, the ability of E2 to induce an LH surge depends on the presence and activation of progesterone [44]. However, progesterone only increases in a narrow window before ovulation, about 12 h before the LH spike [45], and causes LH and FSH to increase, later leading to ovulation. Progesterone is an essential mediator of ovulation as it affects the regulation of the genes located in the granulosa cells of the ovulatory follicles, for example, the encoding proteases ADAMST1 and CTSL. Proteolytic enzymes digest the proteins of the follicle wall and help in its disintegration during ovulation [46].

On the other hand, if the progesterone concentration is constantly above the physiological level that triggers the LH surge, such as during the luteal phase, when taking contraceptives, or during pregnancy, it desensitizes the body’s receptors and/or GnRH. In such a situation, the accumulation of LH and its release are impossible and ovulation does not occur [47]. Hence, raising the progesterone level in the body a few days before ovulation may result in its complete blockage, which is what probably happened in the patient’s previous cycles.

Ovulation is an event influenced by many endogenous and exogenous factors. Not only the regulated work of the HPO axis, neuro-endocrine economy, or cytokine balance in the ovary determine the success of this key reproductive process [46]. A disturbed body metabolism, chronic inflammation, autoimmunity, and a deficiency of vitamins and microelements, cofactors of genetic changes and proliferative factors, as well as the deprivation of biological rhythms, can all generate ovulation disorders. Hence, multifactorial treatment, lifestyle, and dietary changes increase the chances of restoring spontaneous ovulation among PCOS patients. However, heterogeneous ovarian function and the possibility of ovulation occurring on different days of successive menstrual cycles in the same person should also be taken into account. Additionally, it is in this context that the correct incorporation of progesterone, consistent with the observations of the current menstrual cycle, are of great importance for the success of infertility treatment among women suffering from PCOS [48]. 

Progesterone is a hormone that regulates the key reproductive processes, starting from ovulation via implantation, to placental development and pregnancy [49]. The administration of progesterone to patients with PCOS makes great clinical sense due to the pleiotropic effect of this hormone on the body. It not only restores regular bleeding but also reduces hyperandrogenism and insulin resistance. In addition, it has solid immunomodulatory properties that go beyond the reproductive system, affecting the immune processes in the entire body. In various tissues, it can act as a modulator of an inflammatory or anti-inflammatory response [50]. Ovulation disorders in PCOS generate a chronic progesterone deficiency with a supposed excess of E2 in the body, which triggers a whole cascade of adverse neuroendocrine changes [51]. However, through excessive estrogen activity in PCOS, various autoantibodies may be formed, which in turn may contribute to the development of inflammation in the ovarian tissue and, in a “vicious circle”, can maintain the pathomechanism that triggers PCOS [52].

We realize that, ultimately, to confirm our concept, it would be best to conduct randomized cross-over studies where the “gold standard” is randomization, consisting, for instance, of randomly assigning patients to specific study groups. However, a severe limitation here is that a group of patients struggling with infertility expect an immediate effect, also because of the suffering of not being able to conceive a child [53]. Patients with infertility, aware that the compensation of hormonal, metabolic, and immunological disorders of the body that lead to improved fertility is associated with a long-term time factor, do not agree to participate in such studies as they are afraid of extending the duration of their therapy if they were to be put into random research groups and only be given a placebo at first.

Too early an inclusion of progesterone, inadequate to the course of the current menstrual cycle of a given patient, may block ovulation, which, as a result of the therapy of detected disorders, may spontaneously return in women with PCOS. For this reason, we present one of the many clinical cases we see in our team’s daily clinical practice of treating couples with infertility. By including progesterone after a BBT rise and cervical mucus change, both this patient and others are successfully achieving pregnancy in combination with complex, multifaceted infertility treatment.

## 5. Conclusions

One of the clinical problems encountered in polycystic ovary syndrome (PCOS) is luteal phase failure. Many patients have progesterone administrated on a commonly designated day of the cycle. This procedure does not consider the large cycle-to-cycle variations in the actual day of ovulation. The administration of progesterone before ovulation does not improve fertility but may in fact perpetuate menstrual disorders in patients. In this article, we present how, by simply taking into account the self-observations of certain fertility markers, we successfully improved fertility and made conception possible. The proposed clinical case is only a representative example and an illustration of a possible therapy approach. We routinely apply the principles of progesterone administration that have been presented in this manuscript to patients with luteal phase failure being treated for infertility.

## Data Availability

Data supporting the reported results can be obtained from the corresponding author upon any reasonable request.
